# Activation of a synapse weakening pathway by human Val66 but not Met66 pro-brain-derived neurotrophic factor (proBDNF)

**DOI:** 10.1016/j.phrs.2015.12.008

**Published:** 2016-02

**Authors:** Sumangali Kailainathan, Thomas M. Piers, Jee Hyun Yi, Seongmin Choi, Mark S. Fahey, Eva Borger, Frank J. Gunn-Moore, Laurie O’Neill, Michael Lever, Daniel J. Whitcomb, Kwangwook Cho, Shelley J. Allen

**Affiliations:** a Henry Wellcome Laboratories for Integrative Neuroscience and Endocrinology, School of Clinical Sciences, Faculty of Health Sciences, University of Bristol, Whitson Street, Bristol BS1 3NY, UK; b Centre for Synaptic Plasticity, University of Bristol, Whitson Street, Bristol BS1 3NY, UK; c Chonnam-Bristol Frontier Laboratory, Biomedical Research Institute, Chonnam National University Hospital, Gwangju 501-757, South Korea; d Medical and Biological Sciences Building, University of St. Andrews, Fife KY16 9TF, UK; e Learning & Research, School of Clinical Sciences, Faculty of Health Sciences, Southmead Hospital, Bristol BS10 5NB, UK

**Keywords:** JC-1 dye, 5′,6,6′-tetrachloro-1,1′,3,3′-tetraethylbenzimidazolylcarbocyanine iodide, AD, Alzheimer’s disease, ANOVA, analysis of variance, aCSF, artificial cerebrospinal fluid, BDNF, brain-derived neurotrophic factor, CD, circular dichroism, DIV, days *in vitro*, DTT, dithiothreitol, ECD, extracellular domain, fEPSP, field excitatory postsynaptic potentials, FCR, furin cleavage-resistant, GSK3β, glycogen synthase kinase 3β, IPTG, isopropyl β-D-1-thiogalactopyranoside, LDH, lactate dehydrogenase, LTD, long-term depression, LTP, long-term potentiation, LD, luminal domain, NAD, nicotinamide adenine dinucleotide, p75NTR, pan-neurotrophin receptor, PMS, phenazinemethosulfate, PONDR, predictor of naturally disordered regions, RU, response units, SNP, single nucleotide polymorphism, SDS PAGE, sodium dodecyl sulfate polyacrylamide electrophoresis, s.e.m., standard error of mean, SPR, surface plasmon resonance, TrkBIg_2_, TrkB immunoglobulin-like domain 2, TrkB, tyrosine kinase B, WT, wild-type, Pro-brain-derived neurotrophic factor (proBDNF), Val66Met polymorphism, Long-term depression (LTD), Binding kinetics, Neurotrophin receptors, Chemical compounds studied in this article

## Abstract

This study describes a fundamental functional difference between the two main polymorphisms of the pro-form of brain-derived neurotrophic factor (proBDNF), providing an explanation as to why these forms have such different age-related neurological outcomes. Healthy young carriers of the Met66 form (present in ∼30% Caucasians) have reduced hippocampal volume and impaired hippocampal-dependent memory function, yet the same polymorphic population shows enhanced cognitive recovery after traumatic brain injury, delayed cognitive dysfunction during aging, and lower risk of late-onset Alzheimer’s disease (AD) compared to those with the more common Val66 polymorphism. To examine the differences between the protein polymorphisms in structure, kinetics of binding to proBDNF receptors and *in vitro* function, we generated purified cleavage-resistant human variants. Intriguingly, we found no statistical differences in those characteristics. As anticipated, exogenous application of proBDNF Val66 to rat hippocampal slices dysregulated synaptic plasticity, inhibiting long-term potentiation (LTP) and facilitating long-term depression (LTD). We subsequently observed that this occurred *via* the glycogen synthase kinase 3β (GSK3β) activation pathway. However, surprisingly, we found that Met66 had no such effects on either LTP or LTD. These novel findings suggest that, unlike Val66, the Met66 variant does not facilitate synapse weakening signaling, perhaps accounting for its protective effects with aging.

## Graphical abstract

**Figure:**
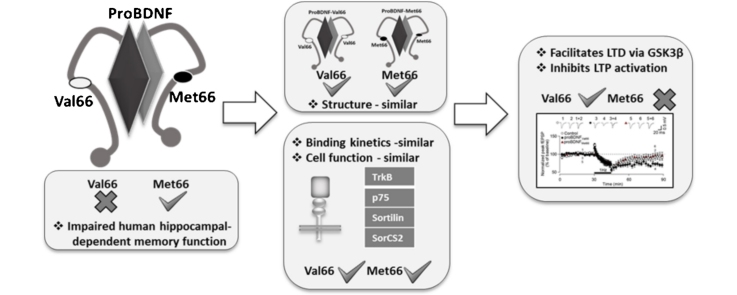

## 1 Introduction

The neurotrophin brain-derived neurotrophic factor (BDNF) is first translated as the precursor form, proBDNF. A common single nucleotide polymorphism (SNP) rs6265 at codon 66 in the pro-domain of human proBDNF changes the amino acid sequence from the canonical sequence of valine (Val66) to methionine (Met66). Whilst generally less prevalent, the Met66 polymorphism can be found in ∼50% Asians, ∼30% Caucasian and ∼4% African–Americans [1]. Interestingly, population studies show healthy young adult Met66 carriers to have a significant volumetric reduction in specific brain structures including the hippocampus [2], [3], [4] and are known to suffer from hippocampal-dependent memory deficits [2], [5]. Intriguingly however, studies also show a slower overall cognitive decline with increasing age in healthy proBDNF Met66 carriers [4], [5], [6], [7] compared with Val66. Furthermore, Met66 carriers have increased recovery of high order executive functioning [8] and general cognitive functions [9] after traumatic brain injury. Accordingly, evidence would suggest that Met66 confers some neuroprotective effects that are not present with Val66. Exactly why this is the case and the mechanisms responsible, however, remain to be fully explained.

BDNF mediates neurotrophic functions including neuronal survival and differentiation, mainly *via* its specific receptor, tyrosine kinase B (TrkB) [10], [11]. In contrast, studies have shown that the common proBDNF Val66 predominantly facilitates unfavorable signaling such as growth cone collapse [12], [13], reduction in spine density [14], [15] and apoptosis [14], [16], [17], [18], [19] through its pan-neurotrophin receptor (p75NTR) and its co-receptors sortilin or the sortilin-related VPS10 domain family member SorCS2. Synaptic plasticity, widely considered to be a cellular correlate for learning and memory in the brain, is also differentially regulated by BDNF and proBDNF Val66; whilst BDNF is important in the induction of long-term potentiation (LTP) and synapse strengthening [20], by contrast, proBDNF Val66 facilitates long-term depression (LTD) and the degradation of synapses [15], [21]. Accordingly, proBDNF Val66 appears to directly modulate synapse function by promoting synapse weakening. The relationship between proBDNF Met66 and synapse function, however, remains to be fully characterized.

Earlier studies showed that the presence of one Met66 allele is sufficient to impair trafficking of the proBDNF Met66 variant to the distal dendrites, leading to a reduction in both activity-dependent secretion and synaptic targeting of BDNF-containing vesicles in neurons [2], [22], [23]. Indeed, *in vitro* electrophysiological studies of Met66 homozygous knock-in mice showed a selective impairment in activity-dependent synaptic plasticity [24]. However, these changes seen in Met66 mice could be attributed to the reduced hippocampal BDNF levels associated with this polymorphism [25], and it is yet to be determined whether these observed impairments are actually due, instead, to differences in binding to the receptors p75NTR [16], sortilin [21] and SorCS2 [12] and/or intracellular neuronal signaling induced by the proBDNF Val66 and Met66 variants.

The present study aimed to analyze and compare the structure of the proBDNF variants, their receptor binding dynamics, neuronal intracellular signaling, and capacity to regulate LTP and LTD. Although previous studies have examined the Met66 variant in knock-in mice [24], here we purified two cleavage-resistant human proBDNF variants and used the proteins in exogenous application assays. Doing so allowed us to exclude any potential endogenous differences in protein secretion and indirect BDNF signaling, as well as avoid possible unknown genetic factors present in knock-in models. Constructs of proBDNF were produced in their non-glycosylated forms in *Escherichia coli*. Others [14] report no functional difference between proBDNF obtained from baculovirus and *E. coli*. Even though we cannot completely exclude the influence of post-translational modifications on protein structure and function, in this study the Val66 and Met66 polymorphisms were expressed, purified and treated identically throughout and are compared on this basis.

## 2 Methods

### 2.1 Sub-cloning of proBDNF expression constructs

Human proBDNF DNA constructs were produced as proteolytically cleavable wild-type (WT) and furin cleavage-resistant (FCR) forms: (with mutation for FCR): ^127^RR^128^ to ^127^AA^128,^ numbering as for human proBDNF canonical isoform 1 (ExPASy bioinformatics protein identifier, P23560-1). ProBDNF Met66 was formed from Val66 DNA construct by site directed mutagenesis (QuikChange^®^ Site-Directed Mutagenesis Kit, Stratagene). Genomic DNA was used as a template to generate proBDNF constructs without the signal sequence and devoid of extraneous tags. Sub-cloning was into pET 24a(+) (Novagen) with transformation into XL1-Blue *E. coli* (Stratagene).

### 2.2 ProBDNF protein expression, refolding and purification

ProBDNF expression was performed with modifications to the method previously described for proNGF [26], pET 24a(+) plasmids encoding for proBDNF WT and FCR were transformed into BL21 (DE3) RIPL *E. coli* (Stratagene). ProBDNF expression was induced using 1 mM IPTG (isopropyl β-D-1-thiogalactopyranoside) and cells grown for 3 h at 37 °C and then harvested by centrifugation. ProBDNF, expressed in inclusion bodies, was isolated by cell lysis using an X-press (Nike hydraulics, Sweden). The inclusion bodies were washed in 100 mM Tris buffer (pH8.0) containing in succession, 100 mM NaCl and 2.5% (v/v) protease cocktail inhibitor (Sigma # P2174), 1 M NaCl, 1% Triton X-100 and finally 100 mM NaCl. Inclusion bodies were solubilized and purified with modifications to the previously described method for proNGF [27]. Solubilization was carried out in 6 M guanidine hydrochloride containing 50 mM dithiothreitol (DTT), 50 mM Tris pH8.0 and 1 mM EDTA. The solubilized proteins were refolded by rapid dilution into 100 mM Tris pH8.5, 750 mM l-arginine, 5 mM EDTA, 5 mM reduced glutathione and 2 mM oxidized glutathione; samples were then incubated for 4 h at 10 °C. This was dialyzed against buffer containing 50 mM sodium phosphate pH7.2, 1 mM EDTA and 0.05% (v/v) protease cocktail inhibitor at 4 °C.

### 2.3 TrkB immunoglobulin-like domain 2 (TrkBIg_2_) protein expression and purification

TrkBIg_2_ was expressed, purified and refolded as previously described [27].

### 2.4 Preparation of lysates, sodium dodecyl sulfate polyacrylamide electrophoresis (SDS PAGE) analysis and western blotting

Hippocampi pre-incubated with proBDNF were microdissected to isolate the CA1 dendritic-rich region, as previously described [28], and resolved on SDS PAGE. Primary antibodies were anti-BDNF N20 (Santa Cruz, #sc-546); anti-phosphoserine 9 GSK3β (Cell Signaling, #9336); anti-GSK3β (Santa Cruz, #sc-9166); anti-pan tau (Tau5, Invitrogen #AHB0042); anti-phosphorylated tau (PHF-1, kindly provided by Professor Peter Davies, Feinstein Institute, NY); anti-β-actin (AbCam, #ab6276).

### 2.5 Acidic native PAGE analysis

BDNF and proBDNF are basic proteins with high iso-electric points (pI) of 8.5–9.6 and were analyzed on 15% acidic native PAGE to determine the presence of oligomers and soluble aggregates. Gels were stained with Coomassie Blue to visualize the bands.

### 2.6 Furin digestion

Furin cleaves proBDNF specifically at the dibasic cleavage site R-X-(K/R)-R [29]. ProBDNF variants in 100 mM 4-(2-hydroxyethyl)-1-piperazine ethanesulfonic acid (HEPES) pH7.5 and 1 mM CaCl_2_ buffer were digested with 2 units of furin (New England Biolabs, #P8077) at 30 °C for 1 h. Samples were resolved on SDS PAGE and analyzed by western blotting.

### 2.7 Circular dichroism (CD)

Secondary structures of proBDNF variants were evaluated by Far-UV (195–260 nm) CD analysis. Data acquisition was outsourced to Department of Chemistry, University of Warwick, Coventry, UK and was carried out by Dr. Vincent Hall and Professor Alison Rodger using a Jasco J-815 spectrometer. Spectra were recorded in 0.2 nm increments at 100 nm/ min scanning speed with response time of 1 s. Data were acquired using a 1 mm path length cuvette, accumulating eight scans per protein. Mean residue molar CD (Δε; moles amino acids^−1^ dm^3^ cm^−1^) were plotted as a function of wavelength (nm).

### 2.8 Surface plasmon resonance (SPR)

SPR was performed on a Biacore T200 (GE Healthcare), as previously described [27] to determine the kinetic profiles of proBDNF variants to their receptor binding domains TrkBIg_2_ [27], p75NTR extracellular domain (p75NTR ECD) (Sino Biologicals Inc., #3184-H08H), sortilin luminal domain (LD) (R&D systems, #3154-ST-050) and SorCS2 LD (R&D systems, #4238-SR-050). The ligands TrkBIg_2_ (17.8 ± 0.6 fmol/mm^2^), p75NTR ECD (40.5 ± 1.8 fmol/mm^2^), sortilin LD (38.8 ± 1.1 fmol/mm^2^) and SorCS2 LD (32.35 fmol/mm^2^) were immobilized on CM5-S Series sensor chips (GE Healthcare, # BR-1005-30) using the amine-coupling method according to the manufacturer’s instructions. The proBDNF variants (analytes) at a range of 0, 0.625, 1.25, 2.5, 5, 10, 20 nM were allowed to flow over the chip surface in running buffer (10 mM HEPES, 150 mM NaCl, 3 mM EDTA, 0.05% (v/v) surfactant P20 at a final pH7.4). Regeneration of the chip surface was carried out using 10 mM Glycine pH 1.5. Kinetic analyzes (*n *≥ 3) were performed by fitting the kinetic plots to the Langmuir 1:1 mathematical model using Biacore T200 evaluation software. All data were double-referenced and plotted as a function of time. Double referencing included subtraction of the inactive reference surface SPR response units (RU) from the ligand-analyte RU and subtraction of buffer association SPR RU at 0 nM analyte on the activated flow cell from the ligand-analyte responses.

### 2.9 Primary cell culture and lactate dehydrogenase (LDH) assay

Dissociated cortical neurons from CD1 mice embryonic day 16 (E16) were prepared as previously described [30]. Cells were plated in neurobasal media with B27 supplement at 1.25 × 10^5^ cells/cm^2^ on poly-d-lysine coated 96-well plates and maintained at 37 °C in a humidified 5% CO_2_ incubator until DIV11. Cortical neurons were incubated in serum free media for 2 h and subsequently treated with proBDNF variants (0, 0.05–10 nM) for 18 h at 37 °C, and LDH release measured. Cell culture supernatant was transferred into 96-well plates and LDH assay reagent (0.2 M Tris pH8.2, 5 mg/ml lactic acid and 1.3 mg/ ml reaction mix containing 0.35 mg tetrazolium, 0.09 mg phenazine methosulfate (PMS) and 0.9 mg nicotinamide adenine dinucleotide (NAD)) was added, incubated at 37 °C for 10 min and the absorbance measured using a FLUOstar OPTIMA plate reader at 485 nm.

### 2.10 Primary cell culture and JC-1 assay

E16 mouse cortical neurons (DIV11) were used to assess mitochondrial membrane potential in response to proBDNF. Cortical cells cultured in 96-well plates were treated with purified proBDNF WT Val66 and Met66 (0, 0.05, 0.1, 1, 5, 10 nM). Each treatment was carried out in triplicate and the plates were incubated for 18 h at 37 °C. Following incubation, the 96-well plates were processed by the 5′,6,6′-tetrachloro-1,1′,3,3′-tetraethyl benzimidazolylcarbocyanine iodide (JC-1 dye) assay to measure mitochondrial potential. The relative fluorescence of J-aggregates was measured using the FLUOstar OPTIMA plate reader, indicating change in mitochondrial membrane potential.

### 2.11 Animals

All procedures involving animals were carried out in accordance with the UK Animals (Scientific Procedures) Act, 1986 and associated guidelines. Male Wistar rats (Charles River, UK) were used to prepare acute hippocampal slices (P24–26). Rats were housed four or five per cage and allowed access to water and food *ad libitum*. The cages were maintained at a constant temperature (23 ± 1 °C) and relative humidity (60 ± 10%) under a 12 h light/dark cycle (lights on from 07:30 to 19:30).

### 2.12 Slice preparation

Animals were killed by cervical dislocation and decapitation. Following this, the brain was rapidly removed and placed into ice-cold artificial cerebrospinal fluid (aCSF; continuously bubbled with 95% O_2_/5% CO_2_) containing 124 mM NaCl, 3 mM KCl, 26 mM NaHCO_3_, 1.25 mM NaH_2_PO_4_, 2 mM CaCl_2_, 1 mM MgSO_4_, and 10 mM d-glucose. Hippocampi were extracted and transverse hippocampal slices (400 μm thickness) were cut using a McIlwain tissue chopper (Mickle Laboratory Engineering Co.). Following manual separation, the slices were then submerged in aCSF for a minimum of 1 h for recovery before experiments commenced. Slices were pre-incubated with 1 nM proBDNF Val66 or Met66 in 5 ml aCSF for 90 min.

### 2.13 Electrophysiology

Following pre-incubation, field recordings were measured at 30 °C with continuous perfusion with 1 nM proBDNF (3 ml/min). LTP was induced by delivering two tetanic stimuli to the Schaffer collateral input (CA3-CA1 pathway) of acute hippocampus slices. NMDAR-dependent LTD was induced by delivering LFS (1 Hz, 900 pulses, 15 min) at the CA3-CA1 pathway [28]. Field excitatory postsynaptic potentials (fEPSP) were measured in the hippocampal CA1 region using recording electrodes filled with 3 M NaCl. The data acquisition and analysis was performed using WinLTP (www.winltp.com). Briefly, the slope of the evoked fEPSPs was measured, normalized to the pre-conditioning baseline and expressed as a percentage of baseline. The mean values of five consecutive recordings (EPSPs) were calculated at approximately 60 min after the induction of LFS.

### 2.14 Study approval

All animal experiments were carried out in accordance with the UK Scientific Procedures Act, 1986 and associated guidelines; methods were carried out in accordance with the approved guidelines. All experimental protocols were approved by the University of Bristol Animal Welfare & Ethical Review Body.

### 2.15 Statistics

Results were analyzed using Graphpad Prism, v4.0 (GraphPad Software Inc.). All data were given as mean ± standard error of mean (s.e.m.). Comparisons between two experimental groups were performed using unpaired *t*-test (2-tailed). Experimental groups of more than two were compared using one-way analysis of variance (ANOVA) followed by Dunnett’s multiple comparison *post-hoc* test or two-way ANOVA followed by Bonferroni’s *post-hoc* test. Significance was defined as ^*^P < 0.05, ^**^P < 0.01, ^***^P < 0.001.

## 3 Results

### 3.1 No major differences in structure between the proBDNF Val66 and Met66 variants

**Fig. 1:**
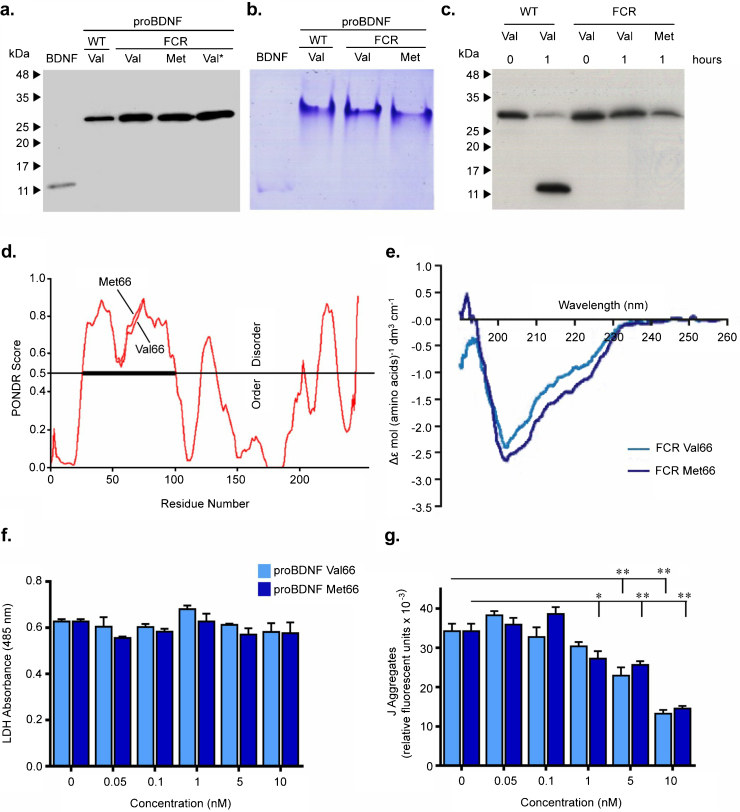
Characterization of proBDNF Val66Met FCR polymorphic variants. (For interpretation of the references to color in this figure legend, the reader is referred to the web version of this article.) (a) Wild-type (WT) proBDNF or furin cleavage-resistant (FCR) proBDNF Val66Met variants (50 ng) and BDNF (10 ng) resolved with SDS PAGE and western blotted using anti-BDNF-N20 1:3000. Val* denotes commercial proBDNF Val66-FCR (Alomone labs). ProBDNF variants have a similar molecular weight of ∼26 kDa (unglycosylated) with high purity. (b) ProBDNF (∼1 μg) resolved on acidic native PAGE gels stained with Coomassie Blue showed no laddering or unresolved proteins, confirming absence of oligomerization and soluble aggregates. Mature BDNF (∼14 kDa) used as standard. (c) ProBDNF variants (50 μg/ml) furin digested 1 h at 30 °C, resolved on 12% SDS PAGE and analyzed using western blotting. Protein bands were detected using anti-BDNF N20 at 1:3000. WT was the positive control, cleaved to mature BDNF. Cleavage-resistant proBDNF variants were intact following furin exposure. (d) Overlay of PONDR outputs of Val66 and Met66. Values are predicted and scaled 0–1. The thick black line (‘disorder bar’) denotes strength of disorder prediction. (e) ProBDNF variants (∼0.1 mg/ml) were measured by CD in the far-UV range. Spectra of both variants showed predominant β-structure with subtle differences in the 222 nm region. Lines show Val66 in light blue Met66 in dark blue. (f) and (g) E16 cortical neurons (DIV11) were serum starved for 2 h and treated with a range of concentrations (0–10 nM) of proBDNF variants for 18 h, after which cells were processed and measured at (f) 485 nm for LDH release (ProBDNF FCR Val66 in light blue, Met66 in dark blue) or (g) at excitation/emission 560/595 nm for JC-1 assay with relative fluorescence of J-aggregates measured (ProBDNF WT Val66 in light blue, Met66 in dark blue). The polymorphism was not associated (*P* = 0.473) with the reduction in membrane potential (two-way ANOVA followed by Bonferroni’s post hoc test). In (f) and (g) changes are represented as column graphs with subsequent analysis using one-way ANOVA followed by Dunnett’s multiple comparison post hoc test for comparison within group. Throughout, error bars denote standard error of mean. Significance is denoted as ^*^P < 0.05, ^**^P < 0.01 and ^***^P < 0.001.

BDNF is first translated in a precursor form as proBDNF and is then N-terminally cleaved by proteases such as furin [29], plasmin [20] and matrix metallo-proteinases [31], [32] to form mature BDNF (∼14 kDa). The proBDNF Val66 and Met66 variants produced in this study resolved similarly on SDS PAGE, showing comparable molecular weight of ∼26 kDa with >95% purity (Fig. 1a). Furthermore, acidic native PAGE gels showed the recombinant proteins to be devoid of oligomerization and aggregation (Fig. 1b) (no aggregation was seen in the stacking gel; supplementary Fig. S1 showing full uncut gel and blots) and proteolytic cleavage using furin showed both cleavage-resistant protein variants to be completely resistant to furin cleavage (Fig. 1c).

Fig. S1 left: Full unedited blots and gels used in Fig. 1a–c. Right: gel or blots trimmed as seen in Fig. 1. Rectangle denotes lanes/treatments shown in edited, cropped representative blots. Uncut version of acidic native gel (molecular weight markers are not used as not applicable for native gels) as seen in Fig. 1b show that the proteins do not aggregate at the top of the gel. All experimental details are as given in Fig. 1.

To determine differences in variant structure, analysis was performed on two fronts: Firstly, *in silico* predictor of naturally disordered regions (PONDR), a web-based neural network [33], [34], [35] and secondly *in vitro* far-UV circular dichroism (CD). The PONDR prediction using the amino acid sequences corresponding to the canonical human proBDNF Val66 isoform 1, P23560-1 (1–247 amino acids) and its variant Met66 polymorphism (VAR_004626) showed Met66 to be more disordered in the region surrounding the Val66Met polymorphism (Fig. 1d). Amino acid residues are predicted here as disordered when the PONDR output value ≥0.5. Far-UV CD analysis showed that overall both variants’ spectra had similar shapes in the far-UV region with a minimum peak at ∼204 nm, indicative of a predominant β-sheet structure (Fig. 1e). Spectral overlay further showed proBDNF Met66 to contain a small increase in negative ellipticity at ∼222 nm, the region characteristic for helical proteins. Thus, limited differences in structure were observed between the two variants.

### 3.2 No significant difference in receptor binding affinities between the Val66 and Met66 proBDNF variants

**Fig. 2:**
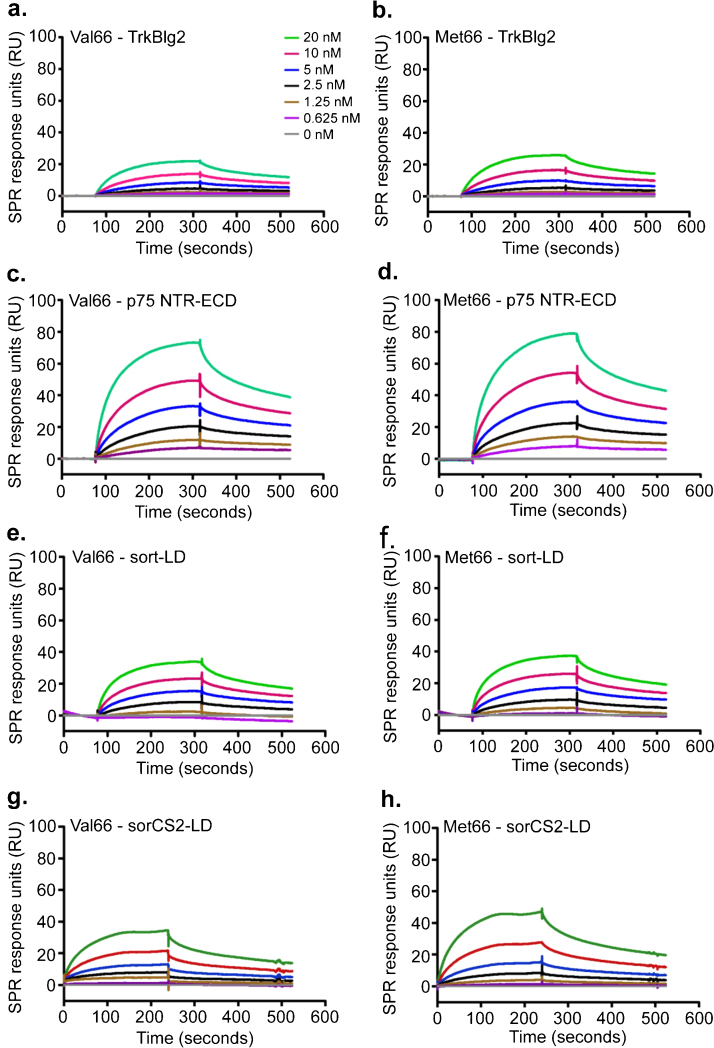
ProBDNF variants are similar in their binding to TrkB, p75NTR, sortilin and SorCS2. Representative sensorgrams are shown for 0–20 nM proBDNF FCR Val66 (a, c, e, and g) and FCR Met66 (b, d, f, and h) binding to their isolated receptor binding domains TrkBIg_2_, p75NTR ECD, sortilin LD and SorCS2 LD. Data was double referenced (RU) and plotted as a function of time. All the kinetic data were fitted to 1:1 Langmuir mathematical model and kinetic constants were calculated. KD denotes mean equilibrium dissociation constant. Mean constants for both Val66 and Met66 variants were not statistically different. Full kinetic constants are given in Table 1.

In the context of the present study, it was important to characterize and compare the binding profiles and kinetic constants of the proBDNF variants to their receptors using surface plasmon resonance (SPR). This would give direct evidence for their comparative ability to bind the tyrosine kinase receptor TrkB, p75NTR, sortilin and SorCS2. Representative SPR sensorgrams are shown in Fig. 2(a–h) and complete on–off rates and equilibrium dissociation constants (*K*_D_) are provided in Table 1. Both variants bound with similar affinity (*K*_D_) as determined by two-tailed *t*-test to the extracellular immunoglobulin-like binding domain of TrkB (TrkBIg_2_) (Val66: 2.32 ± 0.09 nM, *n* = 4; Met66 2.03 ± 0.14 nM, *n* = 3; *P* = 0.780; Fig. 2a and b) and p75NTR extracellular domain (ECD) (Val66: 2.42 ± 0.18 nM, *n* = 5; Met66 2.31 ± 0.22 nM, *n* = 5; *P* = 0.502; Fig. 2c and d). Binding to sortilin luminal domain (LD) (Val66: 2.90 ± 0.37 nM, *n* = 5; Met66: 3.83 ± 0.84 nM, *n* = 6; *P* = 0.312; Fig. 2e and f) and SorCS2 LD (KD Val66 2.23 ± 0.21, *n* = 3; Met66 2.23 ± 0.30 nM, *n* = 3; *P* = 0.614; Fig. 2g and h) also did not differ between the variants. Overall, this is suggestive of little or no difference in the receptor binding affinities, indicating that any difference in cellular effects these variants cause is likely to be due to the differing actions of the proteins themselves.

BDNF was used as a control in each case and the results indicate (see Table 1) that BDNF bound with sub-nanomolar affinity to TrkB and p75NTR while it bound only with low affinity to sortilin and SorCS2.

### 3.3 Significant dose related decrease in mitochondrial membrane potential by both variants but no significant change in LDH release

Previously, cell based assays have shown proBDNF Met66 to bind less well to intracellular sortilin as determined by immunoprecipitation [36]. We were therefore particularly interested to know whether, in cells, there would be a difference between Val66 and Met66 variants with regards to pro-neurotrophin mediated apoptosis, which occurs *via* binding to the extracellular p75NTR–sortilin receptor complex [37]. It seemed possible that the age-related resistance to cognitive decline, which has been associated with the Met66 variant, may be due to an inability of Met66 to bind sortilin at the plasma membrane. Thus, an LDH release assay was initially used to determine the relative effects of the proBDNF variants on the cell death process in serum deprived mouse cortical neurons (DIV11), following treatment for 18 h. However, compared to the untreated serum-deprived cells, treatment with either variant (0.05–10 nM) did not show statistically significant cell death (Fig. 1f). One-way ANOVA followed by Dunnett’s multiple comparison *post hoc* test for comparison within group showed that proBDNF treatment did not affect the release of LDH from cells (Val66: *P *> 0.05; Met66: *P *> 0.05; Fig. 1f). Two-way ANOVA followed by Bonferroni’s multiple comparison *post hoc* test for comparison between groups showed that this was independent of Val66Met substitution (*P* = 0.473).

The effects on change in mitochondrial membrane potential (ψm) were then assessed in serum starved mouse cortical neurons (DIV11) using the JC-1 assay. Treatment with proBDNF (0.05–10 nM) for 18 h showed a significant dose related decrease in mitochondrial membrane potential for both variants as determined by one-way ANOVA followed by Dunnett’s multiple comparison *post hoc* test (Val66: *P* < 0.0001; Met66: *P* < 0.0001; Fig. 1g). However, statistical analysis using two-way ANOVA followed by Bonferroni’s *post hoc* test between the two groups showed the Val66Met polymorphism was not associated (*P* = 0.473) with the reduction in mitochondrial membrane potential. Consistent with the earlier findings, these results suggest no major differences in the fundamental actions of the protein variants.

### 3.4 Inhibition of LTP and facilitation of LTD by Val66 proBDNF variant but not Met66 variant

**Fig. 3:**
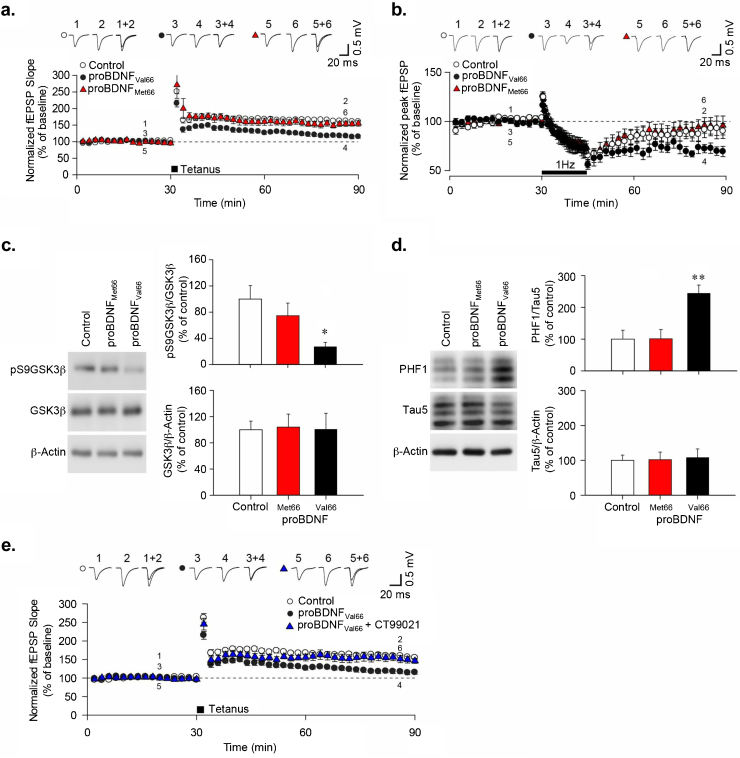
ProBDNF Val66 activates GSK3β to mediate effects on synaptic plasticity. (a) LTP was induced by delivering 2 tetanic stimuli to the Schaffer collateral input of acute hippocampus slices. Prior to tetanus, slices were pre-incubated (90 min) with 1 nM proBDNF Val66 and Met66 FCR variants and perfused constantly with appropriate 1 nM variants. Statistical comparison of the mean fEPSPs values, calculated ∼45 min after application of tetanus, showed prominent differences between Val66 and Met66 in inhibiting LTP. (b) LTD was induced using standard LFS protocol of 1 Hz, 900 pulses for 15 min in rat (P24–26) hippocampal slices. Prior to LFS, slices were pre-incubated (90 min) with 1 nM proBDNF Val66 or Met66 and perfused constantly with 1 nM proBDNF variants as appropriate. Statistical comparison of mean fEPSPs values calculated approximately 45 min after application of LFS showed prominent differences between Val66 and Met66 in facilitating LTD. (c) Western blotting analysis of phosphorylated GSK3β at serine 9 and total levels of GSK3β after incubation with proBDNF variants for 90 min, *n* = 4. (d) Representative western blot showing PHF-1 phosphorylation levels normalized to tau-5 after treatment with proBDNF Val66 or Met66 (1 nM; 90 min). Bar-chart showed quantification of pooled data (*n* = 4). (e) Quantification of LTP levels following 2 tetanic stimuli with proBDNF Val66 in the presence and absence of CT99021 (1 μM; *n* = 6). Significance (^*^P < 0.05; ^**^P < 0.01) was determined using one-way ANOVA with Tukey’s multiple comparison post-hoc test, comparing proBDNF-treated groups to non-treated control groups. Throughout, control experiments were without exposure to proBDNF and error bars denote s.e.m.

The absence of any substantial difference between the two proBDNF variants, either structurally or functionally, was surprising, especially given the widely reported contrast in their effects on cognitive function. Since proBDNF is known to facilitate LTD *via* p75NTR [21], we therefore sought to examine if the exogenous application of the proteins had any consequences on synaptic plasticity, a molecular correlate of learning and memory, in rat hippocampal slices. Application of 1 nM proBDNF Val66 caused a significant inhibition of LTP (control: 164.9 ± 0.66%, *n* = 12; Val66: 119.1 ± 0.79%, *n* = 6; *P* < 0.001), whereas unexpectedly, no such inhibition was observed when tissue was exposed to proBDNF Met66 (162.7 ± 1.44%, *n* = 6; *P* = 0.290; Fig. 3a. We also examined the induction of LTD, as previously investigated [21]. We delivered a 1 Hz low-frequency stimulation protocol, known to induce NMDAR-dependent LTD in juvenile, but not adult, animals [38]. Consistent with this, LTD was not induced in untreated control slices from P24–26 rats (control: 83.80 ± 0.86%, *n* = 6), but was facilitated in slices treated with proBDNF Val66 (Val66: 70.11 ± 0.93%, *n* = 7; Fig. 3b). We hypothesized that the facilitation of LTD by Val66 reflected the activation of LTD signaling. However, surprisingly, exposure to proBDNF Met66 failed to facilitate LTD when compared to the proBDNF Val66 treated slices (95.81 ± 0.58%, *n* = 7; Fig. 3b). The differences seen between the variants and the controls were statistically significant (one-way ANOVA followed by Tukey’s multiple comparison *post-hoc* test, proBDNF Val66 compared to proBDNF Met66 (*P* < 0.001), proBDNF Val66 compared to controls (*P* < 0.001)).

### 3.5 Val66 proBDNF variant specifically activates GSK3β

Since activation of glycogen synthase kinase-3β (GSK3β), a serine/threonine protein kinase, is critical for the induction of NMDAR-dependent LTD [39], we examined the effect of proBDNF variants on GSK3β activation. Exposure of hippocampal slices to proBDNF Val66 resulted in significant dephosphorylation of Ser9-GSK3β, a marker of increased kinase activity, when compared to control levels (control: 100 ± 20.42%; Val66: 27.2 ± 6.74%; *n* = 4; *P* < 0.05; Fig. 3c; Supplementary Fig. S2 shows full uncut blots). However, no significant increase in GSK3β activation was observed when slices were exposed to proBDNF Met66 (74.49 ± 19.36%, *n* = 4; *P* > 0.05; Fig. 3c). These effects were independent of a change in total levels of GSK3β (control: 100 ± 13.13%; Met66: 104.01 ± 19.79%; Val66: 100.65 ± 24.41%; *n* = 4; *P* > 0.05; Fig. 3c). This suggests that whilst proBDNF Val66 can activate GSK3β, the Met66 variant does not, and may go some way to explain their differing effects on synaptic plasticity.

Fig. S2 Full unedited blots used in Fig. 3c and d. Rectangle denotes lanes/treatments shown in edited, cropped representative blots.

### 3.6 ProBDNF Val66 variant inhibits LTP *via* the activation of GSK3β

To test whether proBDNF might mediate its effects on plasticity *via* GSK3β, we exposed hippocampal slices to proBDNF after pre-incubation with the GSK3β inhibitor CT-99021, and tested whether LTP could be induced. Remarkably, CT-99021 prevented the proBDNF Val66-mediated inhibition of LTP (control: 158.35 ± 5.75%, *n* = 6; proBDNF + CT: 156.10 ± 10.46%, *n* = 6; Fig. 3e). This suggests that the actions of proBDNF Val66 on synaptic plasticity are likely mediated through the activation of GSK3β.

### 3.7 Tau phosphorylation induced by Val66 proBDNF variant not apparent with Met66 variant

The presence of hyperphosphorylated tau protein is a neuropathological hallmark of AD pathology [40]. Interestingly, the phosphorylation of tau has recently been identified as a critical step in hippocampal LTD induction [41]. Given that GSK3β is an important regulator of tau phosphorylation, we therefore investigated the regulation of phosphorylation of tau by the two proBDNF variants. We found that the proBDNF Val66 variant significantly increased tau phosphorylation (control: 100 ± 28.19%; Val66: 244.55 ± 25.95%, *P* < 0.01) when compared to control (*n* = 4; Fig. 3d), whilst the proBDNF Met66 variant had no effect (101.16 ± 29.3%, *P* > 0.05 when compared to control, *n* = 4). Together, these data are consistent with a specific effect of the proBDNF Val66 variant on synaptic plasticity, where GSK3β is activated to cause dysregulation of synaptic plasticity.

## 4 Discussion

Previous studies have identified divergent effects in healthy young and older proBDNF Met66 carriers *versus* the Val66 homozygous individuals. Young adult Met66 carriers had reduced cognitive function and hippocampal volume compared with those with Val66 [2], [3], [4], [5], whereas the Met66 allele conferred protection against cognitive decline with aging and after traumatic brain injury [6], [7], [9].

In order to establish whether physical differences between them could account for their contrasting effects in cognitive function, these polymorphic proteins were produced as cleavage-resistant forms and compared directly for the first time for differences in structure, receptor binding kinetics and function. By theoretical analysis, using PONDR, Met66 was seen to be more disordered in the region surrounding the polymorphism. Far-UV CD analysis indicated a predominance of β-sheet structure for both, although Met66 showed a minor difference in a region characteristic for helical proteins. Neither of these factors affected the resistance to furin cleavage; both forms were cleavage-resistant.

We then investigated the possible differences in receptor binding using SPR. The subtle changes seen in CD analysis between proBDNF Val66 and Met66 were not reflected in SPR binding assays, which showed similar binding affinities for both polymorphic variants to their receptors TrkB and p75NTR, most likely as a consequence of those receptors binding to the mature peptide of proBDNF. This too may account for the lack of statistical difference in binding between the proBDNF polymorphisms at TrkB and p75NTR. Pertinent to this, a previous mutagenesis study showed the evolutionarily conserved 103-arginine residue in the mature region of BDNF to be important for both mature and proBDNF to bind TrkB [42]. In addition, recently, proBDNF but not its isolated PRO domain was shown to co-immunoprecipitate with p75NTR [12], suggesting an interaction through the mature domain of proBDNF. However, proBDNF is difficult to crystalize due to its disordered PRO region [12] and its exact binding domains to p75NTR and TrkB are not elucidated. It is also noted that this binding affinity of BDNF and proBDNF to its receptors may not reflect all situations within the neuron due to the formation of p75NTR–TrkB, p75NTR–sortilin and p75NTR–SorCS2 complexes. However, this study compares the Val66 and Met66 forms and in this context there was no difference in binding to individual p75NTR or TrkB receptors. Taken together, then, we believe this is compelling evidence that the proBDNF variants do not differ significantly in terms of fundamental form and mode of action, and therefore any observed differences in terms of downstream signaling effects of the proteins is likely to reflect specific actions of the variants at the functional level.

Because the full-length sortilin receptor, which facilitates the intracellular trafficking of proBDNF, is not amenable to SPR, it was necessary to examine the isolated luminal domain in the present study. Previously, the binding affinity of proBDNF Val66 to its receptor sortilin luminal domain has been reported variably in the literature [16], [43], possibly attributable to the difference in species, cleavage-resistant mutations and post-translational modifications. ProBDNF Met66 variant has been reported to bind less tightly to intracellular sortilin compared to Val66 during biosynthesis and secretion [23], [36] although binding affinities have not been determined. Here, contrary to our expectations, we found no difference in the binding affinities of Val66 and Met66 variants to sortilin using SPR analysis. More recently, SorCS2 has been shown to be a receptor for proBDNF [12], [44]. SPR analysis performed, using SorCS2 in this study did not detect any difference in proBDNF Val66 or Met66 binding. However, this may be due to the use of isolated receptor binding domains in SPR rather than the full-length receptor and/or the role of the presence of p75NTR co-receptor complexes in proBDNF binding.

In an effort to examine the differential effect of the proBDNF variants on apoptosis in a neuronal context we used serum deprived cortical neurons following proBDNF treatment for 18 h. We performed an assay to measure the amount of LDH released into the culture medium by dying or damaged cells with compromised cell membrane integrity. Treatment with either polymorphism up to 10 nM did not increase cell death. However, although previous findings have shown that proBDNF-induced apoptosis occurs *via* binding to sortilin and p75NTR [37], and proBDNF Val66 mediate cell death has been shown in cultured cerebral ganglionic neurons [17] and sympathetic neurons [16], the triggering of apoptosis does not seem to be inevitable in all cells; others found that proBDNF in hippocampal neurons did not induce apoptosis [14]. ProBDNF has also been shown to have some protective effects on neurons [42] making it more difficult to interpret its effects *in vitro*. Therefore cortical neurons were tested for changes in mitochondrial membrane potential, a more subtle form of change, after 18 h. Both variants of proBDNF mediated a reduction in membrane potential. Thus addition of proBDNF shows that it has a negative effect on mitochondrial membrane potential, which may act as a precursor to release of cytochrome C and caspase 3 cleavage, an event upstream of that assessed by the LDH assay. However, both Val66 and Met66 variants had similar effects, suggesting that this was not variant specific.

The possibility that the Met66 variant may differentially affect neuronal plasticity was then tested. As expected, using electrophysiology, the proBDNF Val66 variant was shown to be able to facilitate LTD. Compared to the untreated control slices the facilitation was significant but modest at ∼14%. This was in accord with previous studies performed in mouse hippocampal slices, which used a murine furin cleavage-resistant proBDNF Val66 [21] and in mice overexpressing proBDNF Val66 [15]. However, in contrast with the study by Woo et al. [21] the data here show that proBDNF Val66 was also able to inhibit tetanic stimuli-induced LTP. This apparent contradiction may perhaps be explained by differences between the two experimental approaches, the studies by Woo et al., were performed with mouse proBDNF Val66 cleavage resistant protein coupled to a his-tag, whilst our studies were performed with human proBDNF cleavage resistant protein without tags, but with differing incubation time and concentration. However, the comparable LTD data between the two studies would suggest that any differences in protein utilized are specific to LTP not LTD signalling. The most likely reason is probably therefore the use of rat hippocampus in this study whereas mouse tissue was used in the study by Woo et al. [21].

Very recently, studies have shown that GSK3β mediated tau phosphorylation has an important role in LTD [41], [45] and is reported to be dysregulated in cognitive disorders including AD [46] and a non-pathological model [47]. Furthermore, it has previously been shown that the impairment of synaptic plasticity in an AD and PD pathology model was mediated by GSK3β [48], [49]. Given the activation of GSK3β by the proBDNF Val66 variant specifically, as shown here for the first time, it is proposed that proBDNF mediates its effects on plasticity *via* GSK3β, thus providing a mechanistic link between proBDNF Val66 and LTP inhibition. Together, the data presented here suggest that proBDNF Val66 inhibits LTP through an enhancement of LTD signaling *via* the GSK3β-pTau pathway.

In contrast, we show that proBDNF Met66 has no effect on the GSK3β-pTau signal pathway. Taken together these data provide a convincing picture of a proBDNF Val66 specific effect on hippocampal synaptic plasticity. Here, proBDNF Val66 activates a GSK3β-pTau signaling pathway leading to synapse weakening that is entirely absent in the presence of the common Met66 variant.

This study further adds to the findings of Ninan et al. [24], which showed Met66 knock-in mice to exhibit impaired LTD, by circumvention of the reduction in acute activity dependent release of BDNF seen in these mice. Thus the exogenous application of human proBDNF used in the present study provides new evidence for the Val66Met polymorphism to have direct functional relevance in age-related cognitive impairment. This is in conjunction with the known decrease in the catalytic form of TrkB with age [50] and AD [51] compared to p75NTR [52] which makes likely an increased role of LTD in synaptic plasticity with aging and cognitive impairment. This may explain a bias towards protection against cognitive deficit in later life, of proBDNF Met66, with its reduced ability to facilitate synaptic weakening. This highlights the importance of understanding the activation of synapse weakening signaling in both normal and pathological aging conditions, and the potential impact this may have in the development of novel therapeutic interventions.

## 5 Conclusion

In order to try to explain the age-related functional differences seen in carriers of the common Val66Met polymorphism, the separate human recombinant proteins were produced and the biophysical, kinetic, cellular and synaptic differences between the two common proBDNF polymorphisms were assessed. Theoretical analysis of structure, using PONDR, and analysis of far-UV CD measurement showed only minor structural differences between the polymorphisms. Neither could the polymorphisms be differentiated in terms of their binding kinetics to receptor binding domains. Furthermore, in cell assays, both variants caused a similarly significant dose dependent reduction in membrane potential, although not in release of LDH in cortical neurons. However, differences were noted by electrophysiology, in rat hippocampal slices. ProBDNF Val66 was able to facilitate LTD and inhibit LTP, whereas Met66 was not. Further analysis revealed that Val66 was able to mediate its effects on synaptic plasticity *via* GSK3β, this provides the process of association between Val66 and LTP inhibition. Thus proBDNF Val66 may inhibit LTP by enhancing LTD signaling *via* the GSK3β-pTau pathway. These data also suggest a possible explanation as to why Met66 may confer protection against cognitive deficit in later life, due to its reduced ability to facilitate synaptic weakening.

## Author contributions

S.K., S.J.A., D.W., T.M.P., F.G-M. and K.C. designed and directed different aspects of the study and wrote the paper. M.F. and S.K. carried out protein expression and purification, S.K., T.M.P., J.H.Y., D.W., E.B., S.C., M.L. and L.O’N. carried out experiments. Final Figs. were produced mainly by S.K., T.M.P. and J.H.Y. All authors reviewed the manuscript.

## Figures and Tables

**Table 1:** Summary of proBDNF receptor binding kinetics.

	*n*	(*k*_a _± s.e.m.) × 10^6^ (M^−1^ s^−1^)	(*k*_d_ ± s.e.m.) × 10^−3^ (s^−1^)	(*K*_D_ ± s.e.m.) × 10^−9^ (M)	*P* of *K*_D_ Val66 vs Met66
TrkBIg_2_					
Val66	4	1.61 ± 0.31	3.67 ± 0.52	2.32 ± 0.09	0.780
Met66	3	1.99 ± 0.47	3.92 ± 0.67	2.03 ± 0.14	
BDNF	7	4.57 ± 0.76	2.32 ± 0.43	0.58 ± 0.13	

P75NTR ECD					
Val66	5	1.88 ± 0.48	3.94 ± 0.87	2.42 ± 0.18	0.502
Met66	5	1.55 ± 0.43	3.25 ± 0.48	2.31 ± 0.22	
BDNF	4	4.44 ± 0.68	2.51 ± 0.12	0.62 ± 0.12	

Sortilin LD					
Val66	5	2.48 ± 0.63	7.46 ± 2.27	2.90 ± 0.37	0.312
Met66	6	1.56 ± 0.37	5.04 ± 0.87	3.83 ± 0.84	
BDNF	7	3.80 ± 1.37	21.7 ± 9.01	6.44 ± 1.61	

SorCS2 LD					
Val66	3	1.79 ± 0.23	4.00 ± 0.84	2.23 ± 0.21	0.614
Met66	3	2.135 ± 0.45	4.53 ± 0.36	2.23 ± 0.30	
BDNF	3	Low affinity	Low affinity	Low affinity	
