# Supplementary figures and images for: Activation of a synapse weakening pathway by human Val66 but not Met66 pro-brain-derived neurotrophic factor (proBDNF)
Source: Pharmacol Res. 2016 Feb;104:97–107. doi: 10.1016/j.phrs.2015.12.008 (PMC4773404; doi:10.1016/j.phrs.2015.12.008)

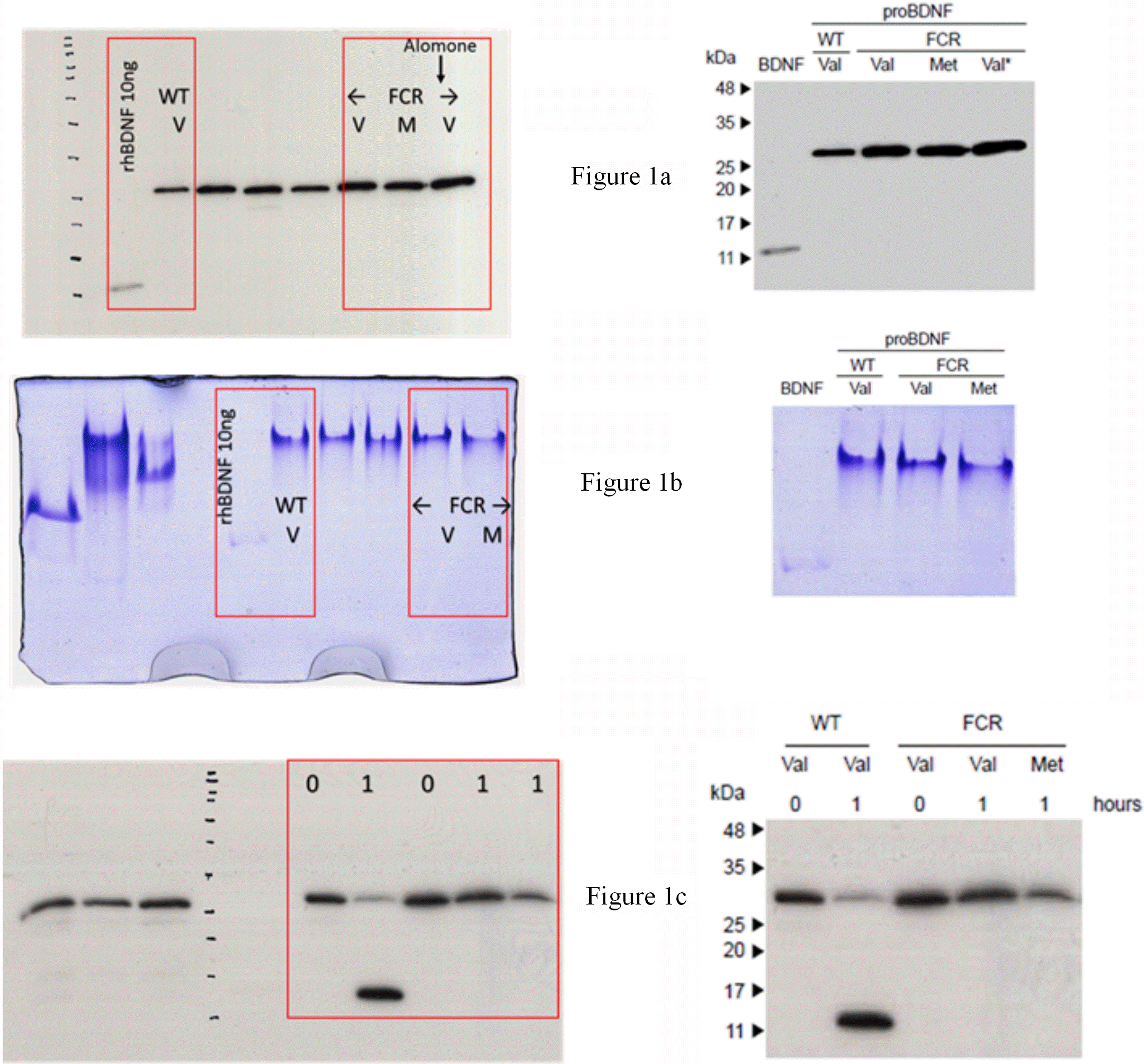

Supplement: Supplementary file 1 — Fig. S1 left: Full unedited blots and gels used in Fig. 1a–c. Right: gel or blots trimmed as seen in Fig. 1. Rectangle denotes lanes/treatments shown in edited, cropped representative blots. Uncut version of acidic native gel (molecular weight markers are not used as not applicable for native gels) as seen in Fig. 1b show that the proteins do not aggregate at the top of the gel. All experimental details are as given in Fig. 1. [file mmc1.jpg]

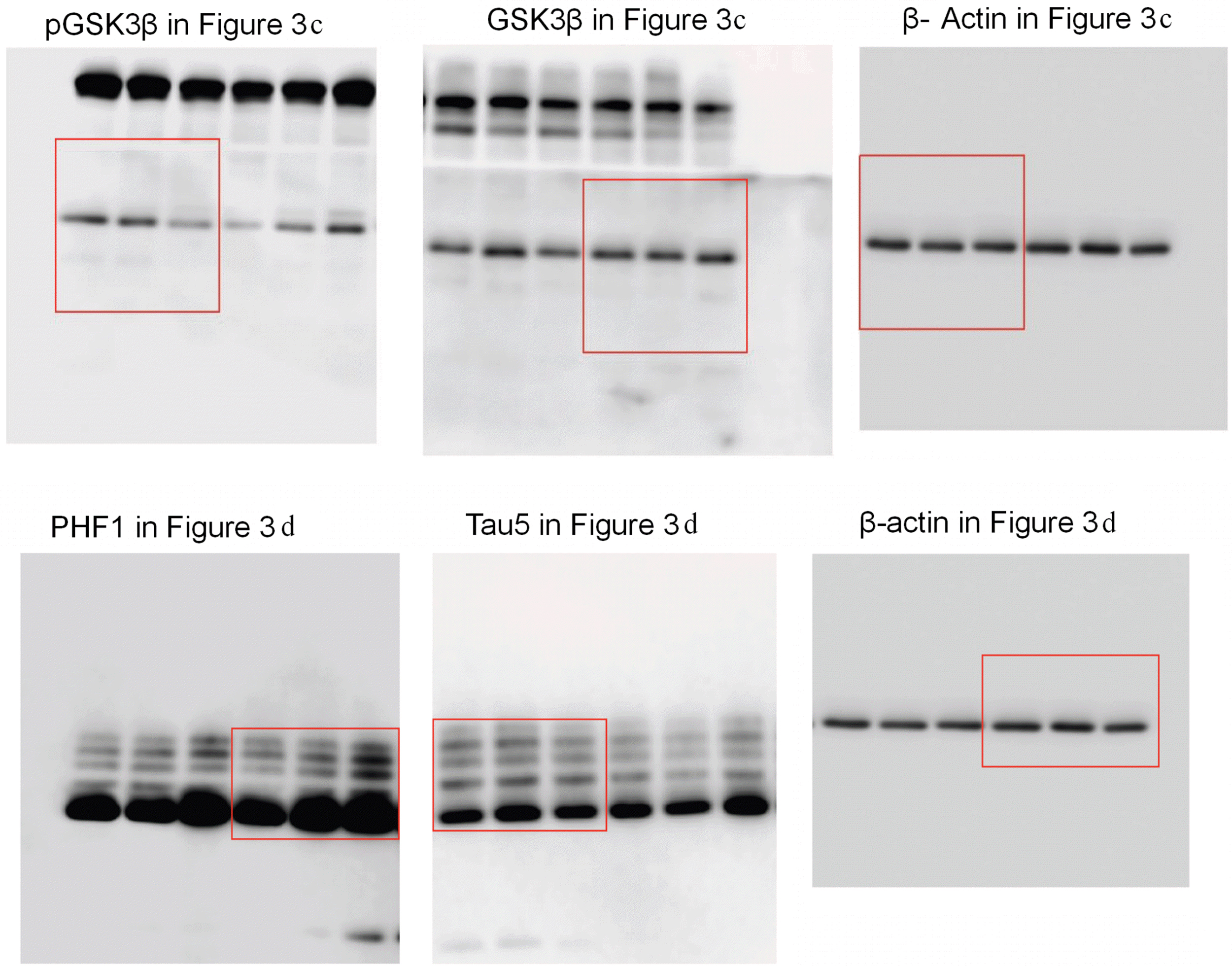

Supplement: Supplementary file 2 — Fig. S2 Full unedited blots used in Fig. 3c and d. Rectangle denotes lanes/treatments shown in edited, cropped representative blots. [file mmc2.jpg]
